# Role of Genetic Variants of Autophagy Genes in Susceptibility for Non-Medullary Thyroid Cancer and Patients Outcome

**DOI:** 10.1371/journal.pone.0094086

**Published:** 2014-04-16

**Authors:** Theo S. Plantinga, Esther van de Vosse, Angelique Huijbers, Mihai G. Netea, Leo A. B. Joosten, Jan W. A. Smit, Romana T. Netea-Maier

**Affiliations:** 1 Department of Internal Medicine, Radboud University Medical Centre, Nijmegen, The Netherlands; 2 Division of Endocrinology, Radboud University Medical Centre, Nijmegen, The Netherlands; 3 Department of Infectious Diseases, Leiden University Medical Center, Leiden, The Netherlands; University of Chicago, United States of America

## Abstract

Autophagy is a central process in regulation of cell survival, cell death and proliferation and plays an important role in carcinogenesis, including thyroid carcinoma. Genetic variation in autophagy components has been demonstrated to influence the capacity to execute autophagy and is associated with disease susceptibility, progression and outcome. In the present study, we assessed whether genetic variation in autophagy genes contributes to susceptibility to develop thyroid carcinoma, disease progression and/or patient outcome. The results indicate that patients carrying the *ATG5* single nucleotide polymorphisms rs2245214 have a higher probability to develop thyroid carcinoma (OR 1.85 (95% CI 1.04–3.23), P = 0.042). In contrast, no significant differences could be observed for the other genetic variants studied in terms of thyroid carcinoma susceptibility. Furthermore, none of the selected genetic variants were associated with clinical parameters of disease progression and outcome. In conclusion, genetic variation in *ATG5*, a central player in the autophagy process, is found to be associated with increased susceptibility for thyroid carcinoma, indicating a role for autophagy in thyroid carcinogenesis.

## Introduction

Epithelial cell derived non-medullary thyroid cancer (NMTC) is the most common endocrine malignancy with a rising incidence during the last decades of which papillary thyroid carcinoma (PTC) and follicular thyroid carcinoma (FTC) represent the vast majority of cases [1]–[3]. Although some tumor-initiating events and susceptibility factors have been identified (radiation exposure, several genetic factors such as genetic rearrarangements or mutations in *RET*, *PTEN* and *APC*) [4], the pathogenesis of NMTC is not completely understood. A better understanding of the underlying molecular mechanisms involved in the development of NMTC could provide diagnostic and prognostic tools and could be a potential source of novel molecular targets for therapy.

Increasing evidence suggests that autophagy plays an important role in the pathophysiology of the malignant process. Autophagy is a complex process of auto-digestion in conditions of cellular stress, hypoxia or energy deprivation. Upon activation, an autophagosome is formed which engulfs cellular components such as organelles, ribosomes and protein aggregates, which are subsequently degraded by fusion of the autophagosome with a lysosome. These degradation products can be reused for building macromolecules and for cellular energy metabolism [5]–[7]. In addition, autophagy has an important role in the regulation of cell death, cell differentiation, induction of cell cycle arrest, and modulation of inflammation [8]. Autophagy may have both preventive and promotional effects on tumorigenesis, which is probably dependent on the type of autophagy initiation, tumor cell type and the stage of tumor development [9], [10]. Hence, it is important to identify the mechanisms that regulate autophagy in malignant transformed cells.

Essential components of the autophagy process are the evolutionary highly conserved ATG proteins, of which more than 30 have currently been identified in yeasts [11], [12]. Common germline genetic variants within genes coding for autophagy components were recently demonstrated to be associated with human disease, ranging from inflammatory bowel disease [13]–[15] to neurodegeneration [16], infectious diseases [17], [18] and allergy [19]. However, despite its central role in cancer initiation and progression, the role of common germline genetic variation within the autophagy system for cancer susceptibility, in particular NMTC, is largely unexplored. Recently, we described that a genetic variant in the autophagy gene *ATG16L1* has an important impact on susceptibility to NMTC [20]. In the present study we broadened the aim of our investigation to assess the potential association of a much broader range of genetic variants in autophagy genes with susceptibility for NMTC, progression and outcome.

## Materials and Methods

### Ethics statement

The study was approved by the Ethical Committee of Radboud University Medical Centre, Nijmegen, The Netherlands. All subjects gave written informed consent. The study has been performed in accordance with the Declaration of Helsinki.

### Thyroid carcinoma patients

All patients with histologically confirmed non-medullary epithelial cell derived NMTC who visited the outpatient clinic at the Division of Endocrinology of the Department of Internal Medicine, Radboud University Medical Centre, Nijmegen, The Netherlands, were asked to participate in genetic testing. The recruitment of the patients took place between November 2009 and June 2010. Primary treatment of the patients consisted of total or near-total thyroidectomy in all of the patients, and modified radical lymph node dissections in patients with confirmed nodal metastases. This was followed by ablation with radioactive iodine (I^131^, RAI) of residual thyroid tissue 4–6 weeks after surgery. If necessary, patients were treated multiple times with RAI to reach remission. Initial cure was defined as undetectable Thyroid Stimulating Hormone stimulated thyroglobulin (Tg) in the absence of anti-Tg antibodies and no evidence of loco-regional disease or distant metastasis on whole body iodine scans (WBS) and/or neck ultrasonography examinations at six to nine months after RAI ablation. Tumor recurrence was defined as new evidence of loco-regional disease or distant metastasis after successful primary therapy. Current disease status was defined as “in remission” in case of undetectable Tg in the absence of anti-Tg antibodies and no evidence of loco-regional disease or distant metastases at the last follow-up visit. Persistent disease status was defined as detectable Tg and/or evidence of loco-regional disease or distant metastases.

Demographic and clinical characteristics (tumor histology and TNM staging), treatment (number of RAI therapy sessions, cumulative RAI dose), follow-up time, the number of re-operations and external beam radiation therapy, if applicable, were retrieved from the patient's medical records (Table 1). The Dutch population based control group consisted of 189 healthy controls (48% women, mean age 61±10 (SD) years) having no evidence of thyroid cancer or other malignancies.

**Table 1:** Clinical, pathological and treatment characteristics of the thyroid carcinoma patient cohort.

Variable	Total (±SD)	Variable	Total (%)
Patients (number)	139	Cum. RAI dose ≤3.7 GBq	35 (25.2%)
Gender (Female/Male)	104/35	Cum. RAI dose 3.8–7.4 GBq	50 (36.0%)
Age at diagnosis, years (mean ± SD)	38.9 (±12.8)	Cum. RAI dose >7.4	54 (38.8%)

Tumor histology	Total (±SD)	TNM staging	Total (%)
Papillary thyroid cancer	99	T1	41 (29.5%)
Follicular thyroid cancer	33	T2	45 (32.3%)
Both papillary and follicular	5	T3	23 (16.5%)
Differentiated thyroid cancer, not further specified	1	T4	11 (8.0%)
Poorly differentiated thyroid cancer	1	Tx	19 (13.7%)
Re-operations	9	N0	72 (51.8%)
External beam radiation therapy	2	N1	46 (33.1%)
Mean duration follow-up, months (mean ± SD)‡	128 (±112)	Nx	21 (15.1%)
RAI sessions 0–1	82 (59.0%)	M0	96 (69.1%)
RAI sessions ≥2	57 (41.0%)	M1	3 (2.1%)
Persistent after ablation	60 (43.2%)	Mx	40 (28.8%)

‡ since diagnosis of NMTC (primary surgery).

### Genotyping

Venous blood was drawn from the cubital vein of all participants into 10 ml EDTA tubes (Monoject). DNA was isolated from whole blood by using the isolation kit Puregene (Gentra Sytems, MN, USA), according to the manufacturer's protocol. Coding non-synonymous single nucleotide polymorphisms (SNPs) and a few SNPs in untranslated regions of the analyzed genes were selected based on previously published associations with human diseases and/or known functional effects on protein function or gene expression. A total of 10 SNPs in *ATG2B*, *ATG5*, *ATG10*, *IRGM*, *LAMP1*, *LAMP3* and *WIPI1* were genotyped (Table 2) with the use of a mass-spectrometry genotyping platform. All SNPs are in Hardy-Weinberg equilibrium in both patient and control groups. Quality control was performed by duplicating samples within and across plates and by the incorporation of positive and negative control samples.

**Table 2:** Genotyped SNPs in genes encoding components of the autophagy machinery.

Gene	SNP ID	Gene region	Amino acid change
***ATG2B***	rs9323945	Exon 19	Asn1124Asp
	rs3759601	Exon 25	Gln1383Glu
***ATG5***	rs2245214	Intron 6	-
***ATG10***	rs3734114	Exon 1	Ser62Pro
	rs1864183	Exon 4	Thr212Met
***IRGM***	rs72553867	Exon 1	Thr94Lys
	rs4958847	3′ UTR	-
***LAMP1***	rs9577229	Exon 3	Ala204Val
***LAMP3***	rs482912	Exon 2	Ile318Val
***WIPI1***	rs883541	Exon 1	Thr31Ile

UTR = untranslated region.

### Statistical analysis

The difference in genotype frequencies between the patients and the control group were analyzed in a dominant, gene dosage and recessive model using logistic regression. The effect of the genotypes on epithelial derived NMTC susceptibility was estimated by calculating odds ratios (ORs) and their 95% confidence intervals (95% CI) using the same statistical methods. We also performed χ^2^ analysis, and if applicable logistic regression, to determine whether tumor size, cumulative RAI dose, number of RAI treatments, disease status after thyroidectomy plus radio-ablation (if applicable) and current disease status were associated with the genotype of the analyzed autophagy genes. The following parameters were analyzed: 1) the tumor size at time of diagnosis was classified according to the 6^th^ edition of the UICC TNM classification [21]; 2) the number of RAI treatments (including RAI ablation) as 0–1 treatments (e.g. no RAI ablation or exclusively ablation of thyroid remnants after (near) total thyroidectomy) or ≥2 treatments; 3) the cumulative RAI dosage as 0–3.7 GBq (0–100 mCi), 3.8–7.4 GBq (101–200 mCi) or >7.4 GBq (>200 mCi); 4) the disease status after ablation as remission or persistent and 5) the current disease status as remission, persistent or recurrent (after previously documented remission).

To test for differences between the three different genotype groups (homozygous wild-type (ancient), heterozygous, homozygous variant (derived)) in mean age at diagnosis, sex distribution or tumor histology (potential confounders), one-way ANOVA and Pearson χ^2^ analysis were used when appropriate. All statistical analyses were carried out with the SPSS software package (version 20.0). Overall, statistical tests were two-sided and a p-value below 0.05 was considered statistically significant.

## Results

### Genetic susceptibility analysis

From all the patients with NMTC who visited the outpatient clinic of the Radboud University Medical Centre, Nijmegen, The Netherlands between November 2009 and June 2010, 139 patients (104 women; mean age 38.9±12.8 (SD) years at time of blood sampling) agreed to participate in the study. The clinical and demographical characteristics of the NMTC patients are summarized in Table 1. No statistically significant differences were found between the patients with different autophagy genetic variant genotypes with respect to the mean age at diagnosis, gender or tumor histology (data not shown).

Statistical analysis of autophagy genetic variants for NMTC susceptibility revealed a statistically significant assocation with the *ATG5* rs2245214 single nucleotide polymorphism. Analysis by applying a dominant model showed an increased risk of the CG/GG genotype for the diagnosis of NMTC compared to the CC genotype (OR = 1.85, P = 0.042), whereas no statistical significance was reached with either a recessive model or a gene dosage model (data not shown). For the other autophagy genetic variants studied, no statistically significant differences were observed concerning susceptibility to develop NMTC with any of the association models tested, i.e. recessive, gene dosage and dominant models (Table 3 and data not shown).

**Table 3:** Genetic distribution of genetic variants in autophagy genes in a cohort of thyroid carcinoma patients (N = 139) and healthy controls (N = 189).

Gene	Polymorphism	Allelic distribution				OR (95% CI)*	P-value*
***ATG2B***	rs9323945		CC	TC		0.65 (0.20–2.18)	0.547
	Asn1124Asp	Patients	133 (96%)	6 (4%)			
		Controls	184 (97%)	5 (3%)			
	rs3759601		CC	GC	GG	0.70 (0.44–1.11)	0.125
	Gln1383Glu	Patients	50 (36%)	67 (48%)	22 (16%)		
		Controls	54 (29%)	105 (55%)	30 (16%)		
***ATG5***	rs2245214		CC	CG	GG	**1.85 (1.04–3.23)**	**0.042**
	Intron 6	Patients	41 (30%)	67 (48%)	31 (22%)		
		Controls	66 (35%)	98 (52%)	25 (13%)		
***ATG10***	rs3734114		CC	TC	TT	1.53 (0.98–2.37)	0.060
	Ser62Pro	Patients	9 (6%)	40 (29%)	90 (65%)		
		Controls	12 (6%)	74 (39%)	103 (55%)		
	rs1864183		AA	GA	GG	1.41 (0.85–2.33)	0.204
	Thr212Met	Patients	32 (23%)	68 (49%)	39 (28%)		
		Controls	46 (24%)	102 (54%)	41 (22%)		
***IRGM***	rs72553867		CC	CA		1.59 (0.76–3.33)	0.256
	Thr94Lys	Patients	124 (89%)	15 (11%)			
		Controls	175 (93%)	14 (7%)			
	rs4958847		AA	GA	GG	0.88 (0.54–1.43)	0.620
	3′ UTR	Patients	1 (1%)	36 (26%)	102 (73%)		
		Controls	3 (2%)	44 (23%)	142 (75%)		
***LAMP1***	rs9577229		CC	TC		0.79 (0.05–12.78)	1.000
	Ala204Val	Patients	138 (99%)	1 (1%)			
		Controls	188 (99%)	1 (1%)			
***LAMP3***	rs482912		AA	GA	GG	0.78 (0.51–1.20)	0.276
	Ile318Val	Patients	11 (8%)	63 (45%)	65 (47%)		
		Controls	18 (10%)	70 (37%)	101 (53%)		
***WIPI1***	rs883541		AA	GA	GG	1.35 (0.88–2.08)	0.185
	Thr31Ile	Patients	74 (53%)	58 (42%)	7 (5%)		
		Controls	115 (61%)	66 (35%)	8 (4%)		

* Dominant model.

### Genotype - phenotype associations

Within the NMTC patient cohort, associations between genotype and tumor size (T stage), number of I^131^ treatments, cumulative I^131^ dose, disease status after ablation and current disease status were assessed using Pearson χ^2^ analysis. For the *ATG5* rs2245214 single nucleotide polymorphism the results are depicted in Table 4. There were no statistically significant differences between the patients in the different genotype groups with respect to TNM staging, number of RAI treatments, cumulative RAI dose and current disease status (Table 4). Furthermore, no associations were observed for any of the other investigated autophagy genetic variants with these clinical parameters (data not shown).

**Table 4:** Summary of *ATG5* rs2245214 genotype in relation to NMTC phenotype association parameters within the NMTC patient group (N = 139).

Variable	CC (%)	CG (%)	GG (%)	Total	P-value‡
**T stage**					0.962
**T1**	13 (31%)	18 (27%)	10 (32%)	41	
**T2**	12 (29%)	22 (33%)	11 (35%)	45	
**T3**	6 (15%)	13 (19%)	4 (13%)	23	
**T4**	4 (10%)	5 (8%)	2 (7%)	11	
**Tx**	6 (15%)	9 (13%)	4 (13%)	19	
**N stage**					0.176
**N0**	23 (56%)	33 (49%)	16 (52%)	72	
**N1**	9 (22%)	27 (40%)	10 (32%)	46	
**Nx**	9 (22%)	7 (11%)	5 (16%)	21	
**M stage**					0.633
**M0**	26 (64%)	49 (73%)	21 (68%)	96	
**M1**	1 (2%)	2 (3%)	0	3	
**Mx**	14 (34%)	16 (24%)	10 (32%)	40	
**RAI treatments (n)** *					0.856
**0–1**	24 (59%)	39 (58%)	19 (61%)	82	
**≥2**	17 (41%)	28 (42%)	12 (39%)	57	
**Cumulative RAI dose (GBq)**					0.626
**≤3.7**	8 (20%)	17 (25%)	10 (32%)	35	
**3.8–7.4**	17 (41%)	22 (33%)	11 (36%)	50	
**>7.4**	16 (39%)	28 (42%)	10 (32%)	54	
**Disease after ablation**					0.872
**Remission**	25 (61%)	37 (55%)	17 (55%)	79	
**Persistent**	16 (39%)	30 (45%)	14 (45%)	60	
**Current disease**					0.230
**Remission**	32 (78%)	48 (72%)	27 (87%)	107	
**Persistent**	7 (17%)	18 (27%)	3 (10%)	28	
**Recurrent**	2 (5%)	1 (1%)	1 (3%)	4	

*Including radio-ablation.

‡ Calculated by Pearson χ^2^ analysis.

## Discussion

The present study was performed to investigate whether common genetic variants in human autophagy genes are associated with NMTC susceptibility, severity and/or clinical outcome. We found that one of the selected genetic variants, the *ATG5* rs2245214 single nucleotide polymorphism, is significantly associated with NMTC susceptibility, but not with NMTC severity or outcome. Furthermore, none of the other selected autophagy SNPs were associated with either susceptibility for NMTC, severity of the disease or clinical outcome.

All of the investigated proteins are involved in the autophagy machinery, some in the early phase of autophagosome formation (ATG2B, ATG5, ATG10, IRGM and WIPI1), the others in the late phase of autophagosome-lysosome fusion (LAMP1 and LAMP3) [22], [23]. In the process of autophagosome formation, ATG5 is recruited to take part in a large protein complex together with ATG12 and ATG16L1 to assemble the double membrane surrounding the autophagic cargo [24], [25]. Autophagy is active at basal levels in all cell types, where it is believed to play a housekeeping role in recycling intracellular components.

In terms of carcinogenesis, the role of autophagy is complex and depends on the type of cancer and the stage of the disease. Defects in autophagy may mediate carcinogenesis through accumulation of protein aggregates and damaged organelles. On the other hand, in apoptotic-competent cells autophagy is cytoprotective, as these cells depend on autophagy to cover their increased energy expenditure [9]. Despite the important role of autophagy for the pathogenesis of cancer, surprisingly little is known about the genetic variation in autophagy genes and its influence on carcinogenesis. In the present study, we assessed the effect of a broad range of genetic variants in autophagy genes for susceptibility to and treatment outcome of differentiated epithelial cell derived NMTC.

The present genetic association study revealed that the G allele of the *ATG5* rs2245214 SNP is associated with increased susceptibility for developing NMTC. In contrast, this *ATG5* SNP was not associated with NMTC severity and outcome as reflected by TNM staging, cumulative RAI dose and disease persistence. The fact that the genetic variants in the other selected autophagy genes are not associated with NMTC susceptibility and severity in our cohort of NMTC patients could indicate that either these proteins have no prominent role in NMTC carcinogenesis or the consequences of the genetic variants for the function of the respective proteins is relatively limited. However, replication studies in other NMTC cohorts should be performed to firmly demonstrate the lack of association of these genetic variants with NMTC susceptibility and severity.

Genetic variation in *ATG5* has previously been linked to systemic lupus erythematosus (same SNP) [26], [27], asthma [19] and neurodegenerative disease [28], indicating the important role of ATG5 in human health and disease. However, the consequences of these genetic variants of *ATG5* for the function of the protein are still unknown and warrant further investigation that should also include previously reported non-autophagic functions of ATG5 [29], [30].

Our previous report of the genetic association of the *ATG16L1* T300A polymorphism (rs2241880) with NMTC susceptibility and severity [20] is now extended by the demonstrated association of the *ATG5* rs2245214 polymorphism with NMTC susceptibility in the present study, confirming the role of autophagy in NMTC pathogenesis. Of note, no additive effects of the two SNPs in *ATG5* and *ATG16L1* were observed, indicating that the two SNPs act independently. Interestingly, both the role of autophagy in NMTC and the therapeutic potential of targeting autophagy for NMTC treatment are confirmed by other studies [10], [31]–[33].

Multiple studies have shown the important role of autophagy in NMTC pathogenesis, representing one of the most prominent downstream pathways of the often aberrantly regulated RAS/RAF/MEK/ERK and PI3K/Akt/mTOR pathways in NMTC, leading to inactivation of the autophagy machinery [34], [35]. In line with these studies, reactivation of autophagy by inhibition of the mTOR kinase results in resensitization of NMTC to chemo- and radiotherapy [33]. In contrast, also opposite effects of signalling through these oncogenes has been described that activate basal autophagy, indicating the complex and context-dependent effects of these pathways on autophagy [36]–[38]. Genetic variants of autophagy genes leading to either less or more functional autophagy machinery could subsequently result in abolished therapy sensitivity and increased carcinogenesis, providing a potential mechanism underlying the observed genetic associations. Additional studies are warranted to dissect the role of autophagy in either promoting or inhibiting carcinogenesis and therapy sensitivity in the context of NMTC subtypes to identify the most effective targeted therapies.

An important point to be considered is that of correction for multiple testing in this study. It has to be taken into account that, when applying correction for multiple testing, statistical significance of the *ATG5* rs2245214 SNP association with NMTC susceptibility is lost. Another limitation that has to be taken into account is the missing data points for the clinical assessment of TNM stageing, which has decreased the statistical power to demonstrate significant differences. The findings obtained in the present study therefore need to be confirmed in larger prospective cohorts in order to draw firm conclusions regarding the definitive role of the genetic polymorphisms described here. Despite of this, it is nevertheless important to observe that the earlier association between *ATG16L1* and NMTC provides indirect support for the findings of the present study.

In conclusion, we have identified the *ATG5* rs2245214 genetic variant as a genetic susceptibility factor in thyroid carcinogenesis. These findings emphasize the therapeutic potential of modulation of ATG5 and ATG16L1, most probably as part of the autophagy machinery, as a novel treatment strategy for NMTC patients.
